# Calcitonin gene-related peptide protects from soluble fms-like tyrosine kinase-1-induced vascular dysfunction in a preeclampsia mouse model

**DOI:** 10.3389/fphys.2023.1221684

**Published:** 2023-08-31

**Authors:** Akansha Mishra, Ancizar Betancourt, Vipin Alukkal Vidyadharan, Chellakkan Selvanesan Blesson, Michael Belfort, Chandra Yallampalli, Madhu Chauhan

**Affiliations:** Department of Obstetrics and Gynecology, Baylor College of Medicine, Houston, TX, United States

**Keywords:** calcitonin gene-related peptide, sFlt-1, mitochondrial function, vascular smooth muscle cells, preeclampsia

## Abstract

**Introduction:** Preeclampsia (PE) is a hypertensive disorder during pregnancy associated with elevated levels of soluble FMS-like tyrosine kinase (sFLT-1) and increased vascular sensitivity to angiotensin II (ATII). Calcitonin gene-related peptide (CALCA) is a potent vasodilator that inhibits the ATII-induced increase in blood pressure and protects against ATII-induced increases in oxidative stress through a mitochondrial-dependent pathway in male mice. In rodent pregnancy, CALCA facilitates pregnancy-induced vascular adaptation. Most of the vascular effects of CALCA are mediated by vascular smooth muscle cells (VSMCs). We recently reported that CALCA treatment inhibits sFLT-1-induced decreases in cAMP synthesis in omental artery smooth muscle cells (OASMCs) isolated from pregnant women and has relaxant effects in omental arteries (OAs) isolated from pregnant women with preeclamptic (PE) pregnancies. The current study was designed to assess the effects of sFLT-1 on mitochondrial bioenergetics in OASMCs isolated from pregnant women in the presence or absence of CALCA and assess the development of vascular dysfunction in sFLT-1 using a mouse model of PE pregnancy.

**Methods:** OASMCs were isolated from pregnant women to assess the effects of sFLT-1 on mitochondrial bioenergetics and oxidative stress using the Seahorse assay and quantitative PCR. Pregnant mice overexpressing sFLT-1 via adenoviral delivery were used to assess the effects of CALCA infusion on the sFLT-1-induced increase in blood pressure, ATII hypersensitivity, fetal growth restriction, and the elevated albumin–creatinine ratio. Systemic blood pressure was recorded in conscious, freely moving mice using implantable radio telemetry devices.

**Results:** CALCA inhibited the following sFLT-1-induced effects: 1) increased oxidative stress and the decreased oxygen consumption rate (OCR) in response to maximal respiration and ATP synthesis; 2) increases in the expression of mitochondrial enzyme complexes in OASMCs; 3) increased mitochondrial fragmentation in OASMCs; 4) decreased expression of mitophagy-associated PINK1 and DRAM1 mRNA expression in OASMCs; and 5) increased blood pressure, ATII hypersensitivity, fetal growth restriction, and the albumin–creatinine ratio in sFLT-1-overexpressing pregnant mice.

**Conclusion:** CALCA inhibits sFLT-1-induced alterations in mitochondrial bioenergetics in vascular smooth muscle cells and development of maternal vascular dysfunction in a mouse model of PE.

## Introduction

Preeclampsia (PE) is a leading cause of maternal and fetal mortality and morbidity and is a major cause of preterm birth (Roberts and Lain, 1998; Sibai, 2006). Despite the increasing research interest to discover new therapeutic approaches to prevent and treat preeclampsia, options remain limited. PE is characterized by hypertension and vascular dysfunction during pregnancy (Filipek and Jurewicz, 2018; Rana et al., 2019; Turbeville and Sasser, 2020), but the pathogenesis of PE is multi-factorial and remains unclear. However, PE is associated with elevated levels of soluble fms-like tyrosine kinase-1 (sFLT-1)-mediated angiotensin II (ATII) hypersensitivity (Burke et al., 2016) with a growing body of evidence of increased oxidative stress and mitochondrial dysfunction (Aouache et al., 2018; Taysi et al., 2019; Marin et al., 2020; Vangrieken et al., 2021a; Huang et al., 2021; Smith et al., 2021). The placenta from PE patients secretes increased sFLT-1 and displays vacuolated mitochondria, indicative of increased oxidative stress damage and reactive oxygen species (ROS) generation, leading to impaired electron transport machinery of mitochondria (Boveris and Chance, 1973; Nohl and Jordan, 1980). In normal pregnancy, physiological mitochondrial ROS production is balanced by antioxidant defense, which is impaired in PE due to decreased antioxidant defense, resulting in damaging effects of oxidative stress (Valko et al., 2007; Zhou et al., 2016; Checa and Aran, 2020). Soluble FLT-1-induced hypertension and vascular dysfunction in PE are associated with impaired mitophagy due to elevated oxidative stress (Kim and Byzova, 2014; Schneider et al., 2015; Marin et al., 2020). Increases in sFLT-1 impair cellular bioenergetics of vascular cells that can contribute to increased constriction in maternal vasculature in PE pregnancy (Vaka et al., 2018a; Hu and Zhang, 2021). A recent study showed that sFLT-1 alters cellular metabolism and mitochondrial bioenergetics of endothelial cells and trophoblast cells in PE (Sanchez-Aranguren et al., 2018). However, the effects of sFLT-1 on mitochondrial function in vascular smooth muscle cells remain largely unknown.

Calcitonin gene-related peptide (CALCA), a 37-amino acid peptide (CALCA/CALCA_1-37_), is a potent vasodilator involved in vascular adaptation in rodent pregnancy (Yallampalli et al., 2013; Yallampalli et al., 2014). In human pregnancy, sensitivity of CALCA vasodilation for both the omental artery (OA) and uterine artery (UA) is increased compared to that reported in non-pregnant women (Chauhan et al., 2021; Chauhan et al., 2022). CALCA functions through a G-protein coupled receptor (GPCR) and calcitonin receptor-like receptor (CRLR), in association with its receptor activity modifying protein 1 (RAMP1), and the majority of its vascular actions are mediated through smooth muscle cells (Yallampalli et al., 2013; Yallampalli et al., 2014). Our recent report shows that sFLT-1 decreases cAMP synthesis and disrupts the receptor system for CALCA in OA smooth muscle cells (OASMCs) from women with healthy pregnancies (Holland et al., 2017; Chauhan et al., 2021; Chauhan et al., 2022). In addition, we reported that CALCA_1-37_ treatment results in relaxation of OA segments isolated from PE pregnancy (Chauhan et al., 2021). Therefore, like the reported inhibition of hypertension by CALCA in hypertensive male mice (Aubdool et al., 2017), CALCA appears to render protection from sFLT-1-induced vascular dysfunction and facilitates vascular relaxation in PE pregnancy. Interestingly, and clinically important, is the report showing that MgSO_4_ treatment for PE increases the serum levels of CALCA and upregulates the expression of CRLR and RAMP1 in the placentas of treated women undergoing PE pregnancy (Zhang et al., 1998; Ariza et al., 2009). This suggests a potential physiological role for an intact CALCA system in human pregnancy, which appears to be impaired in PE. Therefore, based on our reports and reports showing that CALCA attenuates ATII-induced ROS-dependent apoptosis in vascular smooth muscle cells (VSMCs) (Luo et al., 2020a; Chauhan et al., 2021; Chauhan et al., 2022) and protects against oxidative stress through a mitochondrial-dependent pathway in male mice (Tullio et al., 2017), we hypothesized that CALCA treatment will protect from sFLT-1-mediated mitochondrial dysfunction in VSMCs during pregnancy and inhibit the development of sFLT-1-induced PE-like symptoms in mice. Therefore, the current study assessed the effects of sFLT-1 treatment in the presence or absence of CALCA on mitochondrial function in OASMCs isolated from women with normal pregnancy and identified if mice that over-express sFLT-1 during pregnancy in the presence of continuous infusion of CALCA are protected from development of PE-like symptoms such as elevated blood pressure (BP), ATII hypersensitivity, fetal growth restriction, and an elevated albumin–creatinine ratio in mice.

## Methods

### Human samples

The Institutional Review Board at the Baylor College of Medicine approved the collection and use of human tissues (protocol number: H-28527). Omental arteries were collected from pregnant patients undergoing cesarean delivery with normal pregnancy after getting their informed consent. Patients were excluded from the study if they had diabetes, fetal anomalies, multi-fetal pregnancy, or any clinical evidence of maternal or fetal infection.

### Isolation and culture of human OASMCs

Omental arteries were cleaned off of omental fat and enzymatically digested, according to the method described by Chauhan et al. (2021). Briefly cleaned arteries were transferred into a 50-ml flask containing 4.0 ml of the enzymatic dissociation mixture: HBSS (Ca^2+^ and Mg^2+^ free) with the addition of 0.2 mM Ca^2+^, 15 mM HEPES buffer (pH 7.3), 0.125 mg/ml elastase, 0.375 mg/ml soybean trypsin inhibitor, 1 mg/ml collagenase, and 2 mg/ml bovine albumin. After incubation at 37°C for 90 min in a gyratory water bath shaker, the tissues were triturated 10 times into a 10-ml plastic syringe with a 16½-gauge needle and passed through a 100-μm nylon mesh to separate the dispersed cells from undigested vessel wall fragments and debris. The filtered suspension was centrifuged in a siliconized conical plastic tube for 5 min at 200 × g, and the pellet was washed once with Dulbecco's modified Eagle medium (DMEM) with all supplements. The cell pellet was suspended in 5 ml of DMEM (high glucose) supplemented with 25 mM HEPES buffer, 2 mM l-glutamine, 100 U/ml penicillin, 100 μg/ml streptomycin, and 10% (vol/vol) heat-inactivated calf serum, and the dispersed cell suspension was aliquoted into a 25-cm^2^ flask. After 18–24 h, the cultures were washed once with HBSS. Cells were cultured in DMEM containing 10% serum with medium changes at 48–72 h intervals. Omental artery SMCs were maintained at 37°C in a humidified incubator in an atmosphere of 95% air and 5% carbon dioxide and studied at sub-confluence (2 days after they had been plated). Before any treatment, cells were rendered quiescent by deprivation of serum and maintained in a serum-free medium for 12 h.

### Mitochondrial stress test

Respirometry of OASMCs was performed with an Agilent Seahorse XF96 respirometer (Agilent Technologies, CA). A total of 15,000 OASMC cells/well were seeded in 96-well plates in respective media containing low glucose and 10% fetal bovine serum (FBS) for 48 h. Cells were then starved in the growth medium containing 2% charcoal FBS for 8 h, followed by overnight treatment with CALCA (10^−8^M) in the presence or absence of sFLT-1 (10 ng/ml). Seahorse assay was performed in the medium supplemented with 1% FBS, 1 M glucose solution, 100 mM pyruvate, and 200 mM glutamine. Measurements were sequentially performed starting from three cycles of 2.5 µM oligomycin, three cycles of 5 µM carbonyl cyanide-4-(trifluoromethoxy) phenylhydrazone (FCCP), and three cycles of 0.5 µM rotenone +0.5 µM antimycin A. ATP production was defined as the difference between the highest oxygen consumption rate (OCR) during the glucose phase and OCR at the end of the oligomycin phase. Maximal respiration was defined as the difference between the highest OCR during the FCCP phase and OCR at the end of the assay. All data were normalized to the DNA content measured by a fluorometric assay using the Hoechst 33258 stain (385 nm excitation and 450 nm emission: Millipore Sigma). Data analyses were performed using Wave software and XF Report Generators (Agilent Technologies, CA).

### Intracellular ROS generation using 2,7-dichlorodihydrofluorescein diacetate (DCFH-DA)

Omental artery SMCs were grown in DMEM (GIBCO) containing 10% FBS. At 80% confluency, cells were starved overnight in serum-free media and treated with sFLT-1 (10 ng/ml) in the presence or absence of CALCA (10^−8^ M) for 24 h, followed by washing with PBS and addition of 500 μl of the DCFH-DA (Sigma-Aldrich, MO). The cells were then incubated at 37°C for 30 min, followed by washing with phosphate-buffered saline (PBS) and addition of 200 μL of the radio immuno-precipitation assay (RIPA) buffer. The cell lysate was centrifuged at 20,000 x g for 10 min at 4°C, and fluorescence of the supernatant was measured at an excitation wavelength of 485 nm and an emission wavelength of 530 nm. Fluorescence intensities were normalized to the respective protein levels in the lysates determined using the BCA assay kit (Pierce Biotechnology, Inc., IL).

Mitochondrial morphology: Omental artery SMCs (OASMCs) were plated on a Lab-Tek chamber in DMEM (GIBCO) containing 10% fetal bovine serum (FBS) at 37°C at 5% CO_2_ for 24 h. Cells were starved overnight in serum-free media and treated with sFLT-1 (10 ng/ml) in the presence or absence of CALCA (10^−8^ M) for 24 h, followed by washing with phosphate-buffered saline (PBS). The cells were then stained with MitoTracker Deep Red (100 nM, Thermo Fisher Scientific, TX) for 30 min at 37°C at 5% CO_2_ and fixed for 15 min at 37°C with 4% paraformaldehyde. Images of cells were captured using an Olympus FluoView 1000 microscope and converted to binary images of mitochondrial particles using NIH ImageJ Macro, as reported previously [39]. Automated morphometry of mitochondrial particles was further performed using NIH ImageJ Macro to obtain the aspect ratio (major axis/minor axis) and form factor (perimeter 2/4 π x area). Fragmented mitochondria were defined as those with aspect ratios below 2.

### Isolation of mRNA and quantitative PCR

Omental artery SMCs were grown in their respective medium containing 10% fetal bovine serum (FBS). At 80% confluency, cells were starved overnight in serum-free media and treated with sFLT-1 (10 ng/ml) in the presence or absence of CALCA (10^−8^ M) for 24 h. After 24 h, RNA was isolated from OASMCs using the RNeasy kit (QIAGEN), according to the manufacturer’s protocol, and cDNA was synthesized using the Superscript IV VILO Master Mix (Thermo Fisher Scientific, TX). Gene expressions were assessed with target-specific primers, as shown in Table 1 using the SYBR Green real-time PCR master mix (Bio-Rad, CA) using the gene-specific primers shown in Table 1. PCR conditions used were 10 min at 95°C for 1 cycle, 15 s at 95°C, 30 s at 60°C, and 15 s at 72°C for 40 cycles, followed by a melt curve analysis (0.5°C/5 s from 60 to 95°C). Efficiency and specificity of primers were tested by using serial dilutions of cDNA and testing the melt curve of the PCR. Target gene expression was normalized with the average of mRNA expression for β-actin and GAPDH. PCR without cDNA served as a negative.

**TABLE 1:** Primer pairs for target genes.

Primer	Forward primer	Reverse primer
β-Actin	5′-ACT​GAC​TAC​CTC​ATG​AAG​AT-3′	5′-CGT​CAT​ACT​CCT​GCT​TGC​TGA​T-3′
GAPDH	5′-GGT​CTC​CTC​TGA​CTT​CAA​CA-3′	5′-AGC​CAA​ATT​CGT​TGT​CAT​AC-3′
18S	5′-TCG​AAC​GTC​TGC​CCT​ATC​AA-3′	5′-ATG​GTA​GGC​ACG​GCG​ACT​A-3′
DRAM1	5′-TCT​TTA​GTG​CTT​GGA​TTG​GTG-3′	5′-ATG​GAC​TGT​AGG​AGC​GTG​TA-3′
PINK1	5′-TGA​ACA​CAA​TGA​GCC​AGG​AG-3′	5′-GTT​GCT​TGG​GAC​CTC​TCT​TG -3′
ND1	5′-GGG​CTA​CTA​CAA​CCC​TTC​GCT-3′	5′-GAG​GCC​TAG​GTT​GAG​GTT​GAC-3′
ND2	5′-CAC​AGA​AGC​TGC​CAT​CAA​GTA-3′	5′-CCG​GAG​AGT​ATA​TTG​TTG​AAG​AG-3′
CYTB	5′-TCA​TCG​ACC​TCC​CCA​CCC​CAT​C-3′	5′-CGT​CTC​GAG​TGA​TGT​GGG​CGA​TT-3′
COI	5′-TCA​TGA​TCA​CGC​CCT​CAT​A-3′	5′-CAT​CGG​GGT​AGT​CCG​AGT​AA-3′
ATPase6	5′-GCC​CTA​GCC​CAC​TTC​TTA​CC-3′	5′-TTA​AGG​CGA​CAG​CGA​TTT​CT-3′
mt-DNA	5′-ACA​CCC​TCC​TAG​CCT​TAC​TAC-3′	5′- GAT ATA GGG TCG AAG CCG C-3′
Nuclear DNA	5′- AGGGTATCTGGGCTCTGG -3′	5′- GGC TGA AAA GCT CCC GAT TAT-3′

### Mitochondrial DNA

Mitochondrial DNA content was measured by comparing mitochondrial gene levels relative to the nuclear gene by real-time PCR in total DNA isolated from OASMCs using the QIAamp DNA mini kit (QIAGEN, CA). Primers used for mitochondrial DNA and nuclear DNA amplification were as shown in Table 1 and reported previously (Pietrocola et al., 2013).

### Animals

All animal procedures and surgical protocols were reviewed and approved by the Institutional Animal Care and Use Committee at Baylor College of Medicine (Protocol: AN-6694). Baylor College of Medicine is accredited by the Association for Assessment and Accreditation of Laboratory Animal Care International. Adult male and female CD-1 [Crl:CD1(ICR)] mice were purchased from Charles River Laboratories. Animals were housed in a temperature and humidity-regulated environment with a 12-h light cycle, followed by implantation of telemetric transducers for blood pressure recordings. For timed pregnancy experiments, virgin female mice (8–12 weeks old) were placed with a stud male mouse overnight. Observation of a copulation plug the following morning was denoted to be gestational day 0.5 (GD 0.5). Plugged female mice were weighed and removed from the stud cage and grouped as per treatments. Pregnant female mice were injected on GD 8.5 with either murine sFLT-1 adenovirus (a kind gift from Dr. Ananth Karumanchi, CA) with or without CALCA or CMV-null adenovirus (Vector Biosystems, United States) diluted in sterile saline via the tail vein. The dose of sFLT-1 adenovirus was titrated based on batch efficacy to provide plasma sFLT1 values ∼160 ng/ml (used in this study), which result in the hypertension phenotype without significant fetal resorption [1]. Mice injected with CMV-null or sFLT-1 adenoviral particles were treated with saline or CALCA_1-37_ using an osmotic mini pump (ALZET, CA) on GD 8.5 for continuous infusion of the saline or peptide (0.1 ug/hr) until GD 17.5, respectively, at the same time as they were injected with sFLT-1 viral particles. Following BP measurements, mice were euthanized, fetal and placental weights were recorded, urine was collected for measuring albumin/creatinine, and mesenteric arteries were collected for vascular studies.

Blood pressure recordings: Non-pregnant mice were anesthetized with a mixture of ketamine (Ketalar; Parke-Davis, NJ) and xylazine (Gemini; Rugby, NY), and telemetric BP transducers (PA-C10 model; Data Sciences, Minnesota) were implanted. Mice were allowed to fully recover from the surgery for 1 week, followed by mating. The day of inserting the copulatory plug was considered pregnancy day 0.5. Blood pressure (BP) data were recorded for 72 h before mating (non-pregnant), and recording was resumed on day 14.5 of pregnancy and recorded until GD 17.5. The recordings were performed for 30 s at 10-min intervals using the Dataquest ART data acquisition system (DSI, Minnesota).

Vascular reactivity studies: Mesenteric arteries (MA) were dissected free of adherent connective tissues. Artery rings of 2 mm length were mounted on a Multi Wire Myograph System (Model 620M; Danish Myo Technology) for isometric tension recording, as previously described [21]. The myograph bath contained physiological saline solution (PSS) at 37°C and was bubbled with 5% CO_2_ and 95% O_2_. To induce contraction, arterial rings were held at an optimal tension of 2.5 mN and then exposed to potassium chloride (80 mM) for 7 min. Cumulative dose–response curves were constructed. Dose contraction responses to ATII (relative to potassium chloride responses) were determined using a concentration range of 10^–10^M to 10^−5^M. Because of the characteristic response of ATII, it was necessary to use one channel each time. The plateau period lasted seconds, which prevented the simultaneous use of two or more channels. MA responses to bradykinin for norepinephrine-mediated contraction were evaluated to confirm endothelial integrity. Artery segments with >50% relaxation by bradykinin were used in the study. Data distribution was determined using the Shapiro–Wilk test. Comparisons between the groups were performed using a t-test. A *p*-value *< 0.05* was considered significant. Human alpha-CALCA_1-37_ was purchased from Phoenix Pharmaceuticals Inc., United States. All other drugs were purchased from Sigma-Aldrich (MO).

### Statistics

All data are presented as mean ± SEM. For vascular studies, KCL-mediated contraction was used as a reference to calculate the percentage of contraction achieved by ATII. An ATII dose–response curve was performed, and 50% of the requisite dose (µmol) to achieve the peak maximal contraction (EC_50_) was compared between the groups. Concentration–response curves of drugs were fitted to a logistic sigmoid relation, and Emax (maximal constriction effect) was calculated using GraphPad Prism. Repeated measures ANOVA was conducted (treatment and time were considered as factors). The Bonferroni *post hoc* test was used for comparisons of dose–response curves between groups. Other experiments comprising three or more groups were compared using one-way ANOVA, followed by the Bonferroni test to compare the significance between various groups. Student’s two-tailed t-test was used for assessing differences between the two groups. Statistical significance was defined as *p < 0.05*.

## Results

### Effects of sFLT-1 treatment in the presence or absence of CALCA on the development of oxidative stress and the bioenergetics profile of OASMCs

**FIGURE 1:**
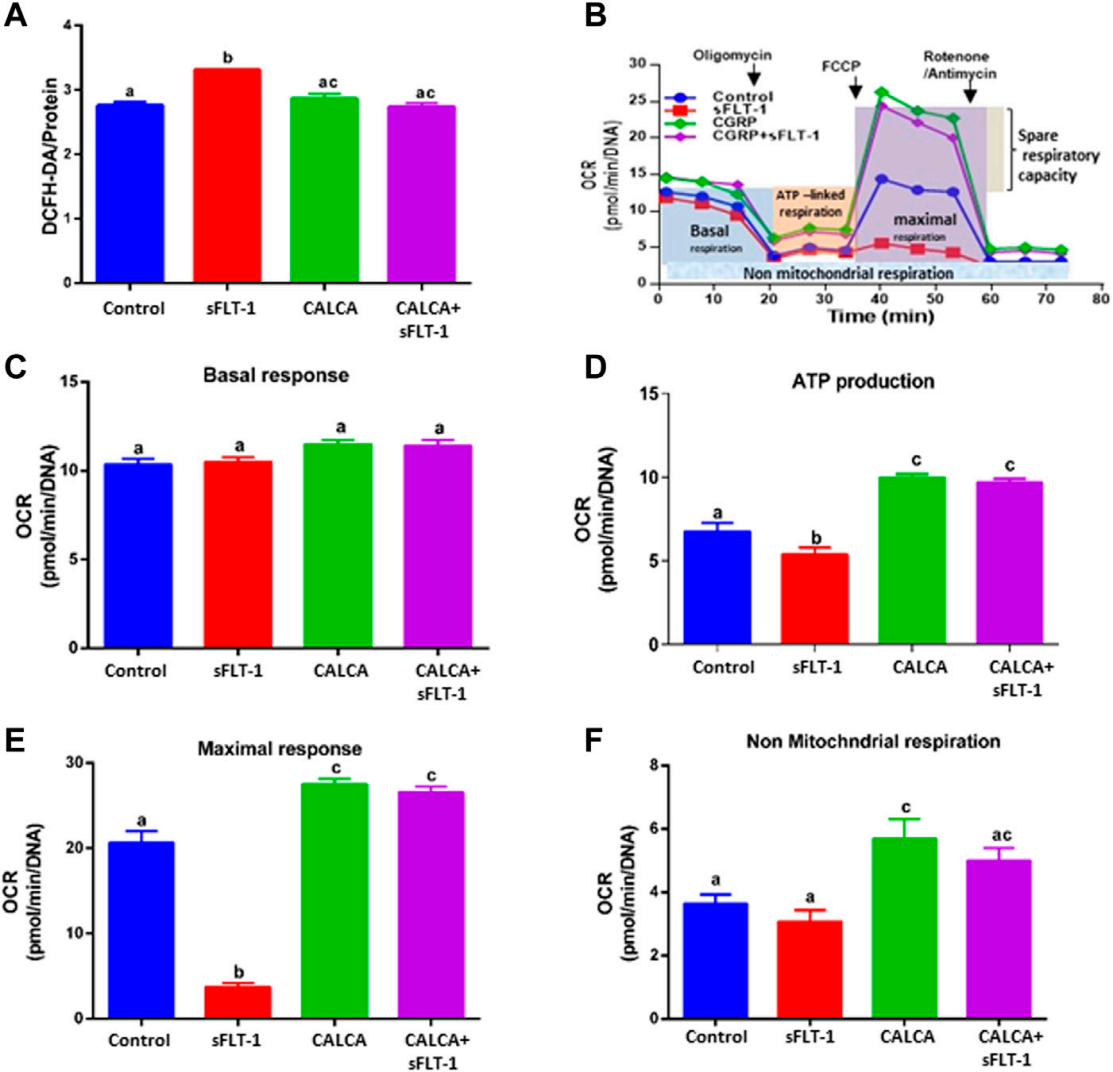
Effects of sFLT-1 treatment in the presence or absence of CALCA on the oxidative stress and bioenergetic profile of omental artery smooth muscle cells. **(A)** Averaged fluorescence of DCFH-DA showing that sFLT-1 increases the oxidative stress in OASMCs, which is inhibited in the presence of CALCA (10^−8^ M; *p < 0.05*). Data were normalized with respective protein concentration and presented in a bar graph as mean ± SEM (*p ≤ 0.05*, n = 3), and **(B)** schematic representation of the bioenergetic profile showing regions that define the basal aerobic respiration, ATP turnover, maximal respiration, spare respiratory capacity, and non-mitochondrial respiration, as measured using a Seahorse XF96 flux analyzer. The figure shows the oxygen consumption rate (OCR) measured before and after the addition of inhibitors (complex-V inhibitor oligomycin, carbonyl cyanide-p-trifluoromethoxyphenyl-hydrazon (FCCP), antimycin A, and rotenone, inhibitors of complex III and I) in cells treated with sFLT-1 in the presence or absence of CALCA; **(C)** the baseline cellular OCR was measured, from which basal respiration was derived by subtracting non-mitochondrial respiration; **(D)** ATP-linked respiration derived by oligomycin treatment and subtracting the oligomycin rate from baseline cellular OCR) oligomycin; **(E)** maximal respiratory capacity derived by subtracting non-mitochondrial respiration from the FCCP rate; **(F)** treatment with antimycin A and rotenone, inhibitors of complex-III, to block the electron transport chain (ETC) function, revealing the non-mitochondrial respiration. Different letters indicate significant difference with *p <* 0.05.

To assess if sFLT-1 alters the levels of cellular ROS in OASMCs and identify if CALCA plays a role in regulating sFLT-1 effects, a live cell ROS indicator (DCFH-DA) was used. Figure 1A shows that sFLT-1 significantly increases ROS in OASMCs compared to the untreated controls. Interestingly, the effects of sFLT-1 on the production of ROS are prevented in the presence of CALCA treatment (*p < 0.01*). The Cell Mito Stress Test was conducted to examine the effect of sFLT-1 treatment on the changes in OCR and mitochondrial function in the presence or absence of CALCA (Figure 1B). Overall analysis using various inhibitors and the algorithm of Seahorse Bioscience Inc. for the Mito Stress Test showed that cells treated with sFLT-1 had significantly lower OCR in response to maximal respiration (*p < 0.01*) along with decreased ATP production (*p < 0.05*) compared to the control in OASMC. However, in the presence of CALCA, sFLT-1-induced decrease in OCR is inhibited (*p < 0.01*). No change was observed in the respiratory reserve with the treatments. Furthermore, CALCA treatment alone shows a significant increase in OCR in response to maximal respiration and ATP production (*p < 0.01*).

### Effects of sFLT-1 treatment on expression of mRNA encoding mitochondrial enzyme complexes in OASMCs in the presence or absence of CALCA treatment

**FIGURE 2:**
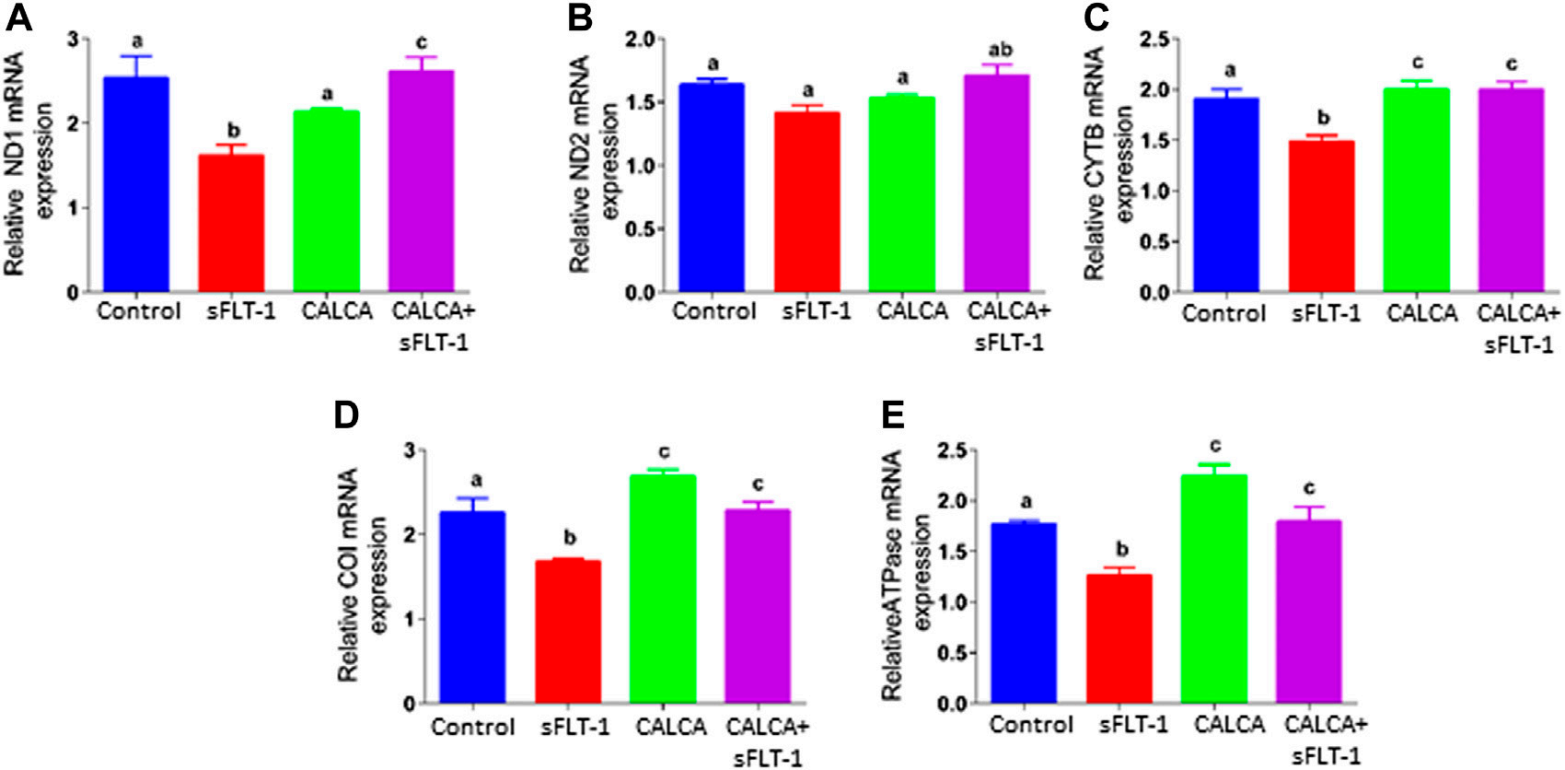
Effects of sFLT-1 treatment in the presence or absence of CALCA on mRNA expression of mitochondrial enzyme complexes. Quantitative PCR showing that sFLT-1-induced decreases in mRNA expression of mitochondrial enzymes encoding mitochondrial complexes ND1 **(A)**, ND2 **(B)**, CYTB **(C)**, COI **(D)**, and ATPase **(E)** in OASMCs. As shown, the effect of sFLT-1 on the expression of ND1 **(A)**, CYTB **(C)**, COI **(D)**, and ATPase **(E)** is inhibited in the presence of CALCA treatment. Expression of the target gene was normalized to the expression of the respective beta-actin mRNA. Data are presented in a bar graph as mean ± SEM. Different letters indicate significant differences. (*p* ≤ *0.05*, n = 3 replicate experiments).

Effects of sFLT-1 (10 ng/ml) treatment on the expression of genes encoding mitochondrial complexes in the presence or absence of CALCA (10^−8^ M) in OASMCs are shown in Figure 2. Figures 2A–E are the quantitative PCR data showing expression of reduced nicotinamide adenine dinucleotide (NADH) dehydrogenase subunits 1(ND1) and ND2 mRNA that encode the proteins involved in mitochondrial complex-I, expression of Cytochrome b (CYTB) mRNA involved in mitochondrial complex-III, expression of Cytochrome c oxidase subunit I (COI) mRNA involved in mitochondrial complex-IV, and expression of ATPase V mRNA involved in mitochondrial complex-V in OASMCs. Data show that sFLT-1 significantly decreases the expression of ND1 (Figure 2A), CYTB (Figure 2C), COI (Figure 2D), and ATPase (Figure 2E) (*p < 0.05*), and the reduced levels of mitochondrial complexes were restored in the presence of CALCA (*p < 0.05*). Furthermore, sFLT-1 treatment shows a trend of decrease in ND2 (Figure 2B) and sFLT-6y1 treatment in the presence of CALCA results in significant upregulation of ND2 mRNA expression compared to the sFLT-1 treatment.

### Effects of sFLT-1 treatment with or without CALCA on mitochondrial morphology and mitochondrial DNA (Mt DNA) content in OASMCs

**FIGURE 3:**
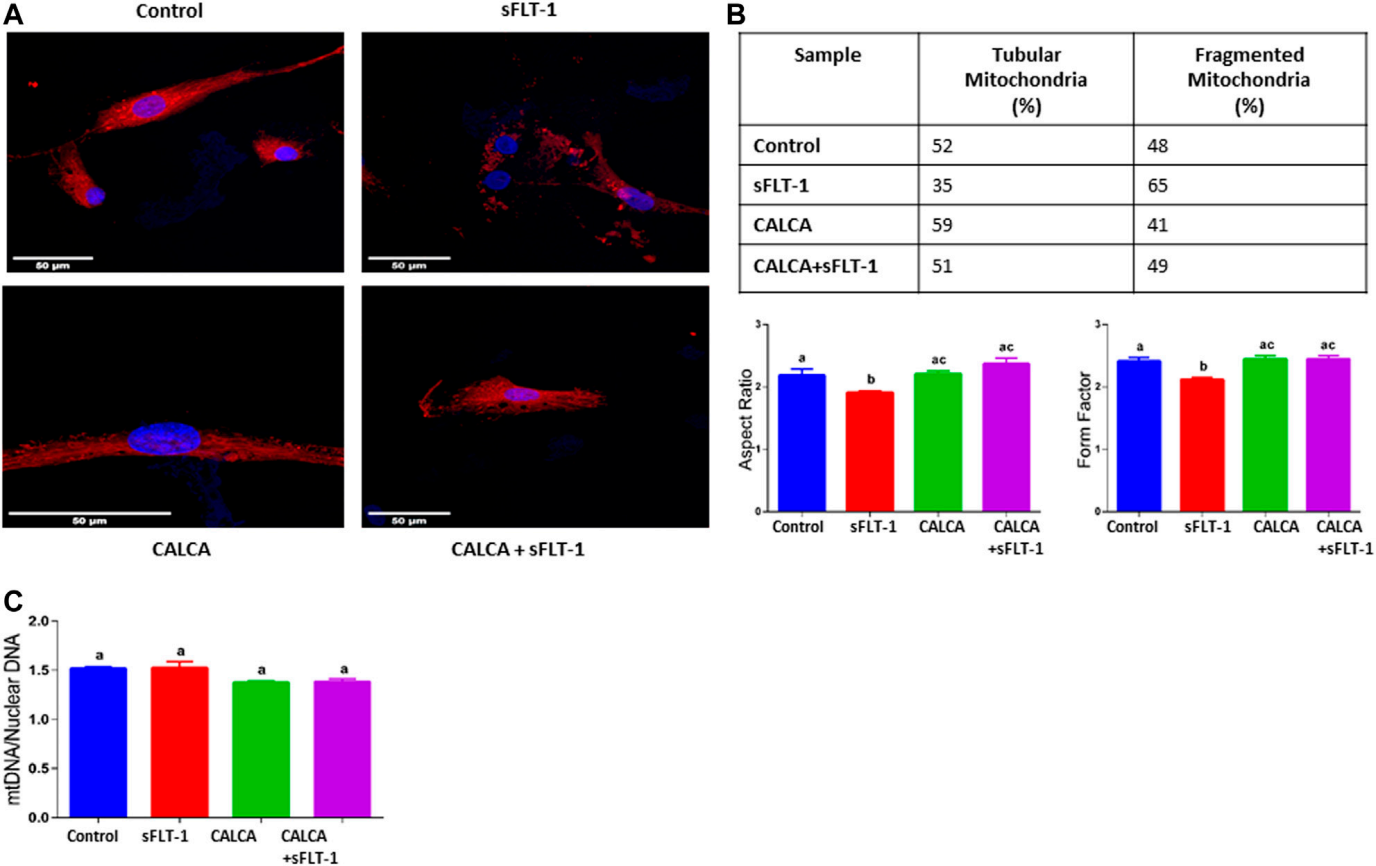
Effects of sFLT-1 treatment on mitochondrial morphology and the mitochondrial DNA content in the presence or absence of CALCA. The figure shows that sFLT-1 treatment causes increased mitochondrial fragmentation, which is inhibited in the presence of CALCA treatment with no effect on the mitochondrial DNA content. **(A)** Representative Mito Tracker Deep Red images of OASMCs treated with sFLT-1 (10 ng/ml) and CALCA (10^−8^M); **(B)** percentage of fragmented mitochondria defined as the aspect ratio below 2 in three independent experiments. Data are presented in bar graphs as mean ± SEM (*p ≤ 0.05*, n = 3) and **(C)** quantitative PCR probed for mitochondrial DNA normalized to nuclear DNA (beta actin). Data are presented in a bar graph as mean ± SEM. (*p ≤ 0.05*, n = 3).

Vulnerability of mitochondria in sFLT-1-treated OASMCs was further noted as accelerated fragmentation of mitochondria (Figures 3A, B). Morphometric analysis showed that the proportion of fragmented mitochondria is higher in sFLT-1-treated cells than that in the controls (Figure 3A). In addition, the aspect ratio and form factor were both decreased in sFLT-1-treated cells, indicating that mitochondria were rounder and fragmented (Figure 3B). Interestingly, the aspect ratio and form factor were both significantly improved when sFLT-1 treatment was performed in the presence of CALCA compared to sFLT-1-alone treatment (*p < 0.05*), indicating a protective effect of CALCA. The mitochondrial (Mt) DNA content measured as Mt DNA/nuclear DNA (mitochondrial to nuclear DNA ratio) is one of the markers for mitochondrial dysfunction. Figure 3C shows that sFLT-1 (10 ng/ml) treatment for 24 h in the presence or absence of CALCA (10^−8^M) has no effect on the mitochondrial/nuclear DNA ratio compared to that in the untreated controls.

### Effects of sFLT-1 treatment with or without CALCA on autophagy-associated genes in OASMCs

**FIGURE 4:**
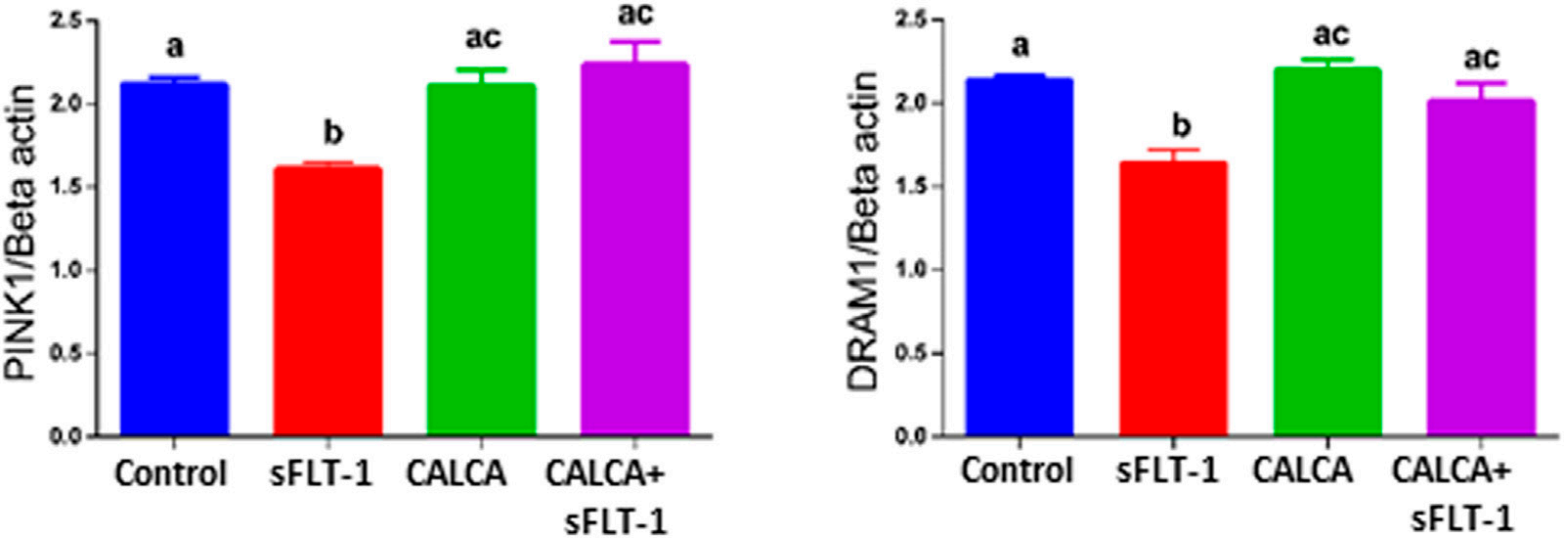
Effects of sFLT-1 treatment on regulators of mitophagy PINK1 and DRAM1. Quantitative PCR data showing the effect of sFLT-1 (10 ng/ml) in the presence or absence of CALCA (10^−8^ M) on mRNA expression of PINK1 and DRAM1. Expression levels of genes are normalized to the mRNA expression of beta-actin. Data are presented in bar graphs as mean ± SEM. (*p ≤ 0.05*, n = 3).

DNA Damage-Regulated Autophagy Modulator 1 (DRAM1) and PTEN-induced kinase 1 (PINK1) are associated with regulation of mitophagy. Expression of DRAM1 is reported to be downregulated in PE pregnancy (Chen et al., 2020). Figure 4 shows that sFLT-1 decreases PINK1 and DRAM1 mRNA expression in OASMCs, and these effects of sFLT-1 are prevented in the presence of CALCA treatment (*p < 0.05*).

### Effect of sFLT-1 treatment on the development of PE-like symptoms in the presence of continuous infusion of CALCA

**FIGURE 5:**
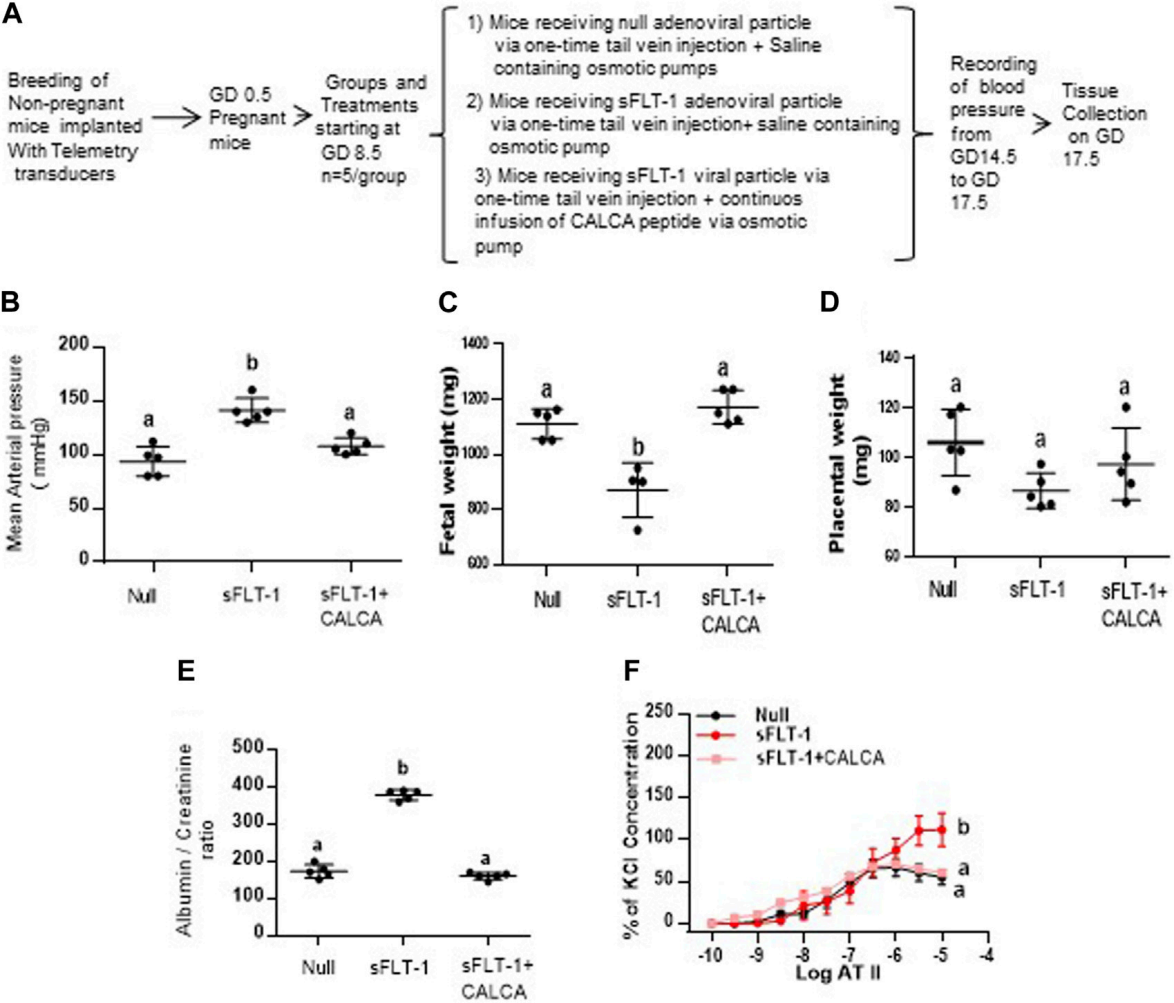
CALCA treatment inhibits development of sFLT-1-induced PE-like symptoms in mice overexpressing sFLT-1 during pregnancy. **(A)** Workflow showing adenoviral-mediated over expression of sFLT-1 in pregnant mice in the presence or absence of CALCA infusion; **(B)** sFLT-1-mediated increase in blood pressure is inhibited in the presence of CALCA treatment (*p ≤ 0.05*, n = 5/group); **(C)** sFLT-1-mediated decrease in fetal growth is inhibited in the presence of CALCA treatment (*p ≤ 0.05*, n = 5/group); **(D)** sFLT-1 treatment shows a trend of decrease in placental weight, which is inhibited in the presence of CALCA (*p > 0.05*); **(E)** sFLT-1-mediated increase in the albumin–creatinine ratio is inhibited in the presence of CALCA treatment (*p ≤ 0.05*, n = 5/group); **(F)** sFLT-1-mediated increase in ATII sensitivity is inhibited in the presence of CALCA treatment (*p ≤ 0.05*, n = 5/group). Different letters indicate significant difference between the groups (*p < 0.05*).

Figure 5 shows the effect of sFLT-1 overexpression in the presence of CALCA infusion in pregnant mice. Figure 5A shows the flowchart showing a group of pregnant mice with all treatments starting at gestational day (GD) 8.5. As shown in Figure 5B, the mean arterial blood pressure is significantly higher in pregnant mice treated with sFLT-1 alone compared to the mice injected with null vector + saline or with sFLT-1 + CALCA infusion. Although Figure 5C shows significant fetal growth retardation in sFLT-1-treated mice, which is inhibited in the group of mice treated with sFLT-1 and CALCA (*p < 0.05*, n = 6), there was a trend of decrease in the placental weight in sFLT-1-treated mice (Figure 5D), which was inhibited in the presence of CALCA treatment. In addition, sFLT-1 treatment increases the albumin–creatinine ratio, which is inhibited in the presence of CALCA infusion (Figure 5E, *p < 0.05,* n = 6/group). In addition, mice treated with sFLT-1 show a significant increase in ATII sensitivity (Figure 5F), and ATII sensitivity is inhibited in the presence of CALCA treatment.

## Discussion

Pregnancy-induced hypertension (PIH), including PE, is one of the main causes of maternal and perinatal mortality. At present, the specific physiological and pathological mechanisms of PIH are not clear (Shirasuna et al., 2015). Due to abnormal placental implantation in PIH, there is increased oxidative stress and release of anti-angiogenic factors such as sFLT-1, leading to maternal hypertension and vasoconstriction. We recently reported that CALCA inhibits sFLT-1-mediated decrease in cAMP synthesis in OASMCs isolated from women with normal pregnancy and relaxes maternal vasculature of women with PE pregnancy (Chauhan et al., 2021). The current study shows that CALCA inhibits sFLT-1-mediated decreases in mitochondrial respiration in response to ATP production and maximal respiration in OASMCs isolated from normal pregnant women. This is supported by data showing that sFLT-1 decreases the mRNA expression of mitochondrial genes *ND1*, *CYTB*, *CO1*, and ATPase in OASMCs and that these decreases are inhibited in the presence of CALCA (*p < 0.05*). In addition, sFLT-1-mediated increases in mitochondrial fragmentation and impaired expression of mitophagy-associated PINK1 and DRAM1 mRNA are prevented in the presence of CALCA in OASMCs (*p < 0.05*). In addition, an *in vivo* study shows that infusion of CALCA inhibits the development of elevated blood pressure, fetal growth restriction, the albumin–creatinine ratio, and ATII sensitivity in mice overexpressing sFLT-1 during pregnancy. Therefore, data from this study suggest that CALCA inhibits sFLT-1-mediated mitochondrial alterations in maternal vasculature and inhibits sFLT-1-induced hypertension and fetal growth restriction in a mouse model of PE pregnancy.

Vascular adaptations such as reduced vascular resistance and arterial pressure that favor a normal pregnancy do not occur in women who develop PE. Instead, severe increases in vascular resistance and arterial pressure are observed due to sFLT-1-mediated vascular dysfunction (Burke et al., 2016; Mayrink et al., 2018). CALCA is a potent vasodilator reported to alleviate hypertension and protect from cardiovascular abnormalities in male mice [23]. Interestingly, circulatory levels of CALCA peptide are downregulated in PE pregnancy (Fei et al., 2012). Our recent report showed that CALCA treatment relaxes omental artery segments isolated from women with PE pregnancy, suggesting a pathophysiological role of CALCA in maternal vasculature in PE pregnancy (Chauhan et al., 2021). Although exciting discoveries in the past two decades have contributed to a better understanding of the molecular basis of PE, mechanisms involved in the pathogenesis of PE are still not well understood.

We reported earlier that sFLT-1 treatment decreases cAMP synthesis in OASMCs isolated from women with normal pregnancy, which is inhibited in the presence of CALCA (Chauhan et al., 2021). In addition, we reported that CALCA causes vascular relaxation in OA of women with PE pregnancy (Chauhan et al., 2021).This suggests a potential role of CALCA in mitigating sFLT-1-mediated vascular dysfunction and facilitating vascular relaxation in PE. PE is a multi-factorial pregnancy disorder with the main manifestations being sFLT-1-induced hypertension and ATII hypersensitivity with increased oxidative stress due to an imbalance between the existing pro-oxidant and antioxidant systems (Loverro et al., 1996; Marin et al., 2020; Smith et al., 2021). Teran et al. showed that women with PE have already-established alterations in the levels of mitochondria targeting antioxidant coenzyme (CoQ10) in the plasma and in the placenta, as compared to normal pregnant women (Teran et al., 2001; Smith et al., 2021). Improved mitochondrial function and ROS are associated with improved blood pressure in sFLT-1-induced hypertensive pregnant rats (Deer et al., 2021). CALCA is shown to protect myocardial cells against oxidative stress through a mitochondrial-dependent pathway, suggesting the involvement of mitochondria in the vascular action of CALCA (Tullio et al., 2017). Figure 1A shows that sFLT-1 alters the cellular ROS level in OASMCs, which is inhibited in the presence of CALCA treatment, suggesting a potential role of CALCA in mitigating sFLT-1-mediated increase in cellular ROS. Mitochondrial homeostasis is crucial for retaining the contractile phenotype of the vascular smooth muscle cells. Using the Seahorse analyzer to perform respirometry assay, the gold standard measurement of the mitochondrial oxidative function with respect to the cumulative activity of all mitochondrial enzyme complexes of the electron transport chain (ETC), this study shows that sFLT-1 does not affect the oxygen consumption rate in the unstimulated OASMCs (Figure 1B) but causes a decrease in the ATP synthesis-linked OCR compared to the basal OCR, which is inhibited in the presence of CALCA. Similarly, the maximal respiratory capacity, as determined by stimulating the cells by injecting uncoupler p-trifluoromethoxy carbonyl cyanide phenyl hydrazone (FCCP), shows that CALCA not only increases the OCR over basal OCR, but it also inhibits sFLT-1-induced decreases in maximal response in OASMCs. It is likely that the increased maximal OCR response induced by CALCA alone is masking the putative prevention of the sFLT-1-induced reduction in OCR.

The electron transport chain in mitochondria is required for all mitochondrial-dependent cell metabolism, and the transfer of electrons from NADH to molecular oxygen to form water is performed by complexes I, III, and IV. The released energy then drives the synthesis of ATP by H^+^-ATPase (complex-V). Therefore, to assess the potential involvement of defects in ETC function by sFLT-1, this study further tested the expression of mRNA that encodes the five multiprotein enzyme complexes that constitute the oxidative phosphorylation (OXPHOS) system of mitochondria. Data show that sFLT-1 decreases the mRNA expression of mitochondrial NADH dehydrogenase, ND1 (involved in complex-I), mitochondrial cytochrome b, CYTB (involved in complex-III), mitochondrial cytochrome oxidase subunit 1 (COI, involved in complex-IV), and mitochondrial ATP synthase/ATPase (involved in complex-V) in OASMCs (Figure 2). Interestingly, CALCA treatment inhibits the decreases in mRNA levels of all enzyme complexes that were targeted by sFLT-1 in OASMCs. Therefore, these data suggest that during normal/unstressed conditions, CALCA plays a potential role in regulating normal OCR, while in the case of stress, such as PE with vascular dysfunction due to elevated sFLT-1 levels, CALCA will facilitate ATP supply and prevent increased stress-induced ATP crisis.

Mitochondria form a highly dynamic intracellular network that executes the “quality control” of the organelle’s population by their fusion, fission, and autophagic degradation (known as ‘mitophagy’) (Green and Reed, 1998; Annesley and Fisher, 2019). Biogenesis of mitochondria and its fusion and fission are crucial regulators of mitochondrial function. Mitochondrial dysfunction has been systematically reported to be involved in the PE pathogenesis in both animal models (Vaka et al., 2018b; Nuzzo et al., 2018) and humans (Mando et al., 2014; Vangrieken et al., 2021b; Vaka et al., 2021). Therefore, this study further assessed the effect of sFLT-1 treatment on fragmentation and elongation (tubular formation) of mitochondria in the presence or absence of CALCA infusion (Figure 3A) in addition to assessing the effect on the copy number (Figure 3C) of mitochondria in OASMCs. Although there is some inconsistency in the literature regarding both the impairment of mitochondrial dynamics and bioenergetics in the setting of PE, this study shows that sFLT-1 treatment decreased the aspect ratio and form factor in OASMCs, which are protected in the presence of CALCA treatment (Figure 3B). Although the number of mtDNA copies per cell can fluctuate with oxidative stress and mitochondrial dysfunction, treatment with sFLT-1 or CALCA had no effect on the mtDNA content in OASMCs (Figure 3C). This suggests a protective role of CALCA in sFLT-1-induced pathological changes in mitochondrial dynamics in PE.

Mitophagy (autophagy in mitochondria) plays a crucial role in mitochondrial quality control (Pietrocola et al., 2013). The regulation of autophagy in PE is an area of research that has been gaining increasing interest with a goal to identify a new target with therapeutic potential. The transducer and activator of transcription (STAT3) are shown to regulate autophagic response and are suggested potential therapeutic targets in PE pathology (Nakashima et al., 2017; Cornelius and Wallace, 2020). Interestingly, CALCA exhibits its antioxidative effect by blocking the STAT3 signaling pathway that is associated with ATII-induced VSMC hypertrophy and hyperplasia (Luo et al., 2020b). Placental expression of two potential regulators of mitophagy, PINK1 (PTEN-induced putative kinase 1) and damage-regulated autophagy modulator (DRAM-1) levels, are deregulated in PE (Chen et al., 2020). Decreases in DRAM1 are reported to contribute to impaired mitochondrial fission and fusion, and its supplementation improves the mitochondrial function and PE symptoms in mice (Chen et al., 2020). However, the effect of sFLT-1 on expression of PINK1 and DRAM1 in VSMC is not known. This study shows that sFLT-1 decreases the mRNA expression of both PINK1 and DRAM-1 mRNA in OASMCs, and these effects of sFLT-1 are prevented in the presence of CALCA (Figure 4). This suggests a potential role of CALCA in regulating impaired autophagy in PE vasculature.

To assess if CALCA treatment can prevent sFLT-1-induced vascular dysfunction *in vivo* in mice, this study further assessed the vascular function in a mouse model of PE that overexpressed sFLT-1 during pregnancy in the presence or absence of CALCA. Interestingly, over-expression of sFLT-1 in the presence of CALCA infusion is protected against increases in BP, ATII hypersensitivity, fetal growth restriction, and the albumin–creatinine ratio compared to the mice treated with sFLT-1 in the absence of CALCA (Figure 5). Circulatory levels of CALCA and its receptors in the placenta are lower in PE compared to the normal healthy pregnancy (Yallampalli et al., 2013; Yallampalli et al., 2014). Therefore, our previous report showing CALCA-mediated relaxation of OA of PE pregnancy and inhibition of the development of PE-like symptoms in PE mice treated with CALCA suggests that the decrease in the endogenous CALCA system in PE pregnancy may serve as a risk factor for sFLT-1-induced maternal vascular dysfunction.

## Conclusion

This study is the first to show that sFLT-1-mediated oxidative stress and mitochondrial dysfunction are protected against in the presence of CALCA in OASMCs isolated from pregnant women, suggesting a functional role of CALCA in mitigating sFLT-1-induced defects in maternal vasculature. In addition, *in vivo* studies in a mouse model of PE demonstrate that CALCA treatment protects from the development of sFLT-1-induced PE-like symptoms such as hypertension, ATII hypersensitivity, the elevated albumin–creatinine ratio, and fetal growth restriction. Taken together, these studies indicate a clinical relevance for the CALCA system in the pathophysiology of sFLT-1-induced hypertension as it occurs in women with PE that imposes a major risk for cardiovascular disease in future and in non-pregnant women with a history of PE (Mongraw-Chaffin et al., 2010; Saxena et al., 2010; Hui and Hladunewich, 2019). This study warrants additional research to further define the role of sFLT-1 in mitochondrial homeostasis in the vasculature of hypertensive pregnancies and identify novel therapeutic targets involving the CALCA system.

## Limitations to the study

The limitation to this study is assessing the effect of CALCA infusion along with administering sFLT-1 viral particles, which demonstrates prevention of sFLT-1-mediated vascular defects in the presence of CALCA. Future studies are warranted to assess the effect of CALCA treatment at different time points in sFLT-1 over-expressing mice to identify if CALCA can reverse the established vascular dysfunction in PE.

## Data Availability

The original contributions presented in the study are included in the article/Supplementary material; further inquiries can be directed to the corresponding authors.
